# Primary prevention of lead poisoning in children: a cross-sectional study to evaluate state specific lead-based paint risk reduction laws in preventing lead poisoning in children

**DOI:** 10.1186/1476-069X-13-93

**Published:** 2014-11-07

**Authors:** Chinaro Kennedy, Robert Lordo, Marissa Scalia Sucosky, Rona Boehm, Mary Jean Brown

**Affiliations:** Centers for Disease Control and Prevention, National Center for Environmental Health/Agency for Toxic Substances and Disease Registry, 4770 Buford Highway, Atlanta, GA 30341 USA; Battelle Memorial Institute, Columbus, OH USA

**Keywords:** Children, Prevention, Law, Lead Poisoning

## Abstract

**Background:**

Children younger than 72 months are most at risk of environmental exposure to lead from ingestion through normal mouthing behavior. Young children are more vulnerable to lead poisoning than adults because lead is absorbed more readily in a child’s gastrointestinal tract. Our focus in this study was to determine the extent to which state mandated lead laws have helped decrease the number of new cases of elevated blood-lead levels (EBLL) in homes where an index case had been identified.

**Methods:**

A cross-sectional study was conducted to compare 682 residential addresses, identified between 2000 and 2009, in two states with and one state without laws to prevent childhood lead poisoning among children younger than 72 months, to determine whether the laws were effective in preventing subsequent cases of lead poisoning detected in residential addresses after the identification of an index case. In this study, childhood lead poisoning was defined as the blood lead level (BLL) that would have triggered an environmental investigation in the residence. The two states with lead laws, Massachusetts (MA) and Ohio (OH), had trigger levels of ≥25 μg/dL and ≥15 μg/dL respectively. In Mississippi (MS), the state without legislation, the trigger level was ≥15 μg/dL.

**Results:**

The two states with lead laws, MA and OH, were 79% less likely than the one without legislation, MS, to have residential addresses with subsequent lead poisoning cases among children younger than 72 months, adjusted OR = 0.21, 95% CI (0.08-0.54).

**Conclusions:**

For the three states studied, the evidence suggests that lead laws such as those studied herein effectively reduced primary exposure to lead among young children living in residential addresses that may have had lead contaminants.

## Background

Children younger than 72 months are most at risk of environmental exposure to lead from ingestion through normal mouthing behavior. Young children are more vulnerable than adults to the deleterious effects of lead poisoning because lead is absorbed more readily in a child’s gastrointestinal tract [1]. Further, the developing nervous system of young children is more vulnerable to the toxic effects of lead [1]. Even at low levels, lead exposure can have a devastating effect on the growth and development of young children. Evidence suggests blood-lead concentrations ≤5 μg/dL are associated with cognitive deficits [2, 3]. From 1991 to 2012, the Centers for Disease Control and Prevention (CDC) defined an elevated blood-lead level (EBLL) as ≥10 μg/dL. However, evidence shows that EBLLs <10 μg/dL can adversely affect the IQ scores of children [4, 5].

In 1971, Massachusetts (MA) passed the MA Childhood Lead Poisoning and Prevention Program (MA Lead Law), a landmark law requiring a program be created to monitor childhood lead poisoning in the state (Table 1). MA was the first state to enact a law specifically tailored to prevent exposure to lead among vulnerable children. The MA Lead Law, as amended, imposes strict penalties on landlords and homeowners who fail to eliminate known sources of lead in dwellings, such as interior paint on walls in poor repair or on surfaces such as window sills and wood work accessible to children. Failure to comply with these laws may result in fines and possible civil liability. According to the law, if a child is diagnosed with lead poisoning, defined as having a BLL ≥25 μg/dL, the owner of the dwelling is liable for civil fines. This rule applies even if the owner is not aware that lead is present in the dwelling. Laws such as the MA Lead Law supplement existing federal requirements that mandate disclosure of lead hazards [6].

**Table 1:** **Summary of Massachusetts and Ohio’s lead laws adapted from:** [6, 7]

Summary of Massachusetts’ ^1^ and Ohio’s ^2^ lead laws			
1971	1987	1993	2004
Massachusetts	Massachusetts	Massachusetts	Ohio
Lead Law passed	Lead Law amended	Lead Law amended again	Lead Law Enacted
• Owners must inspect and delead house or apartment if it was built before 1978 AND a child younger than 72 months lives there.	• Deleaders must be trained and licensed to delead.	• State introduced interim controls to allow owners to delead over a two-year period. Use of encapsulants was approved for deleading.	• Health Department may enter residence of a child who had been identified as having lead poisoning to conduct risk assessment.
	• Residents must be relocated during deleading.		
• The Department of Public Health created a lead-poisoning–prevention program that would be responsible for enforcing these new rules.	• State established financial assistance for deleading, a $1000 state income tax credit and a grant or loan program.	• Safety precautions during deleading were eased when no children were present.	• Permission to enter must be given by the owner or occupant of the residence.
			• If permission to enter the property was not granted, a court order must be obtained.
• Property owners would now be liable for damages if they did not follow the Massachusetts Lead Law and a child was poisoned by the lead.	• Potential purchasers of residential properties must receive notice about the lead law and have an opportunity to have a lead inspection.	• State increased the income tax credit for deleading to $1500 per housing unit, and a new state fund for lead hazards was created.	• If lead hazards were found, a lead-hazard–control order could be issued requiring the owner or occupant to vacate until a clearance examination had been passed.
	• All healthcare providers must test children for lead, and health insurers must cover the costs of this test.	• Owners with Letters of Interim Control or Letter of Compliance could no longer be held liable for damages while the letters were valid. Insurers were required to provide coverage for any negligence claims (short of gross or willful negligence) brought against owners with Letters of Interim Control or Compliance.	Failure to comply with the lead-hazard–control order would require a court order prohibiting occupancy of the residence until the clearance examination had been passed.

^1^MA lead poisoning that triggers an environmental investigation is defined as BLL ≥25 μg/dL.

^2^OH lead poisoning that triggers an environmental investigation is defined as BLL ≥15 μg/dL.

The lead law enacted in Ohio (OH) in 2004 does not impose penalties as strict as the MA lead law (Table 1). The OH law stipulates that when a child is diagnosed with lead poisoning, defined as BLL ≥15 μ/dL, the health department may enter the dwelling thought to be the source of the lead poisoning with the permission of the occupant or owner, in order to conduct a risk assessment. If the occupant or owner does not grant permission to conduct a risk assessment of the dwelling, the health department may obtain a court order to assess the dwelling. If the results of the risk assessment reveal lead hazards, a lead-hazard control order may be issued by the health department; the control order may require that occupants vacate the dwelling until the unit is examined by a certified inspector and deemed cleared of the potential lead-hazard. The owner or manager may choose the method of control for each lead hazard; however, the method must be approved by the health department. If the owner or manager fails or refuses to comply with a lead-hazard control order, the health department shall issue an order prohibiting occupancy of the dwelling until the unit is examined by a certified inspector and is deemed cleared of the potential lead-hazard. Criminal and civil action can be taken if any licensing or work-practice requirements are violated in the course of correcting lead hazards [7]. Our evaluation focused on the effectiveness of lead laws in preventing primary lead exposure among young children. We evaluated the research hypothesis that fewer new cases of lead poisoning among young children will occur in states with lead laws than in states without lead laws.

The strength of our evaluation lies in its capacity to help determine whether lead laws, regardless of stringency, are effective in decreasing lead poisoning among children younger than 72 months in homes where an initial lead poisoning case has been identified. The primary aim was to determine whether the proportion of addresses with subsequent cases of lead poisoning recorded after identification of an index case, was lower in the lead law states, MA and OH, compared to MS. MA and OH were selected because of the differences in the strength of their law, the length of time the laws have been enacted, and each state’s willingness to participate in the study. To adequately evaluate the relative effectiveness of lead laws, we used Mississippi (MS), which has no lead laws, as a control state. At the time of this study, which was conducted between 2009–2012, of the 35 Childhood Lead Poisoning Prevention Programs (CLPPP) under cooperate agreement with the Centers for Disease Control and Prevention’s (CDC) Healthy Homes and Lead Poisoning and Prevention Program (HHLPPP), Mississippi was the only state that did not have either state or local legislation to prevent childhood lead poisoning, but had a high lead screening penetration rate among children <72 months old. Despite not having legislation to prevent lead poisoning and having the highest poverty rate among children from birth to age 5 years, MS had annual screening rates that was similar to other states that had lead legislation and an annual elevated blood lead level (EBLL) rate similar to that of MA and lower than that of OH (Table 2). From 1991 to 2012, CDC defined an EBLL as a BLL ≥10 μg/dL. According to 2009 national estimates obtained from CDC’s Childhood Blood Lead Surveillance System (CBLS), of children tested, the prevalence of EBLL is ≤1% in all three states. Thus, the main focus of this inquiry was to determine the extent to which a lead law likely reduces the number of new cases with EBLL in addresses where an index case was identified.

**Table 2:** **Census level state demographic information**

STATE	% Census tracts with ≥40% of residents aged birth–5 years living below poverty level (2000 census)	% Children tested for blood lead (2009)	% Children with elevated* blood-lead levels (2009)	% Housing units consisting of rental properties (2000 census)	% Housing units built before 1950 (2000 census)	% Housing units built before 1978 (2000 census)
Massachusetts	8.1	48.6	0.43	29.8	42.77	79.22
Ohio	16.7	16.9	1.53	35.7	31.4	77.2
Mississippi	28.1	16.9	0.46	24.9	11.6	59.4

Source: Adapted from [8–10].

*CDC’s definition of elevated BLL in 2009 was BLL ≥10 μg/dL.

## Methods

### Design and data sources

A cross-sectional study was conducted to determine whether addresses in lead law states compared to states without lead laws, were more or less likely to have subsequent cases of lead poisoning after identification of an index case; the odds of addresses in MA and OH, states with lead laws, were compared to the odds of addresses in MS, state without lead laws. At the time of the study, MS was the only state, under cooperate agreement with the CDC, that had no state or local legislation to protect against childhood lead poisoning. Permission to conduct this cross-sectional study was sought from the state Directors of the Childhood Lead Poisoning Prevention Program. All three states agreed to participate and had the resources to contribute to the findings of this cross sectional study. Data for this cross-sectional study were obtained from CBLS records and the files of public health departments in the three states. The CBLS database serves as a central repository of the national blood-lead surveillance data that the state Healthy Homes and Lead Poisoning Prevention Programs (HHLPPP) provide. CDC has supported state and local HHLPPPs that maintain blood-lead test results analyzed by public and private clinical laboratories and perform various other functions. The blood-lead test results and additional case management and environmental data are sent to CDC quarterly.

The CBLS database holds blood-lead data for many children; however, it was not possible to obtain information from grantee files for all children tested in these three states for the study period, from 2000 to 2009. Lead poisoning cases were defined among children younger than 72 months old, with confirmed BLLs that met or exceeded the thresholds that triggered an environmental investigation in that state. In MS and OH, a child’s BLL ≥15 μg/dL triggers a mandatory environmental investigation in the primary residence; in MA, a child’s BLL ≥25 μg/dL triggers an investigation. Confirmation of BLL was based either on a single venous sample or on two capillary samples within a 12-week period. In order to give each lead poisoned case an equal probability of selection into the study, and to avoid any systematic errors in case selection, case records were selected randomly from children younger than 72 months with at least one BLL listed in the CBLS. Random selection occurred in a three-step process. First, case management record numbers were uploaded into a Microsoft Access© (MS Access) data base. Second, MS Access was used to produce a list of randomly generated case management record numbers. From this list, cases were sequentially selected until the desired numbers of records were pulled.

### Definitions

*Index Case:* The first lead poisoned case identified by case managers between 2000 and 2009 recorded at a given address, with no record of other addresses attached to this case, whose BLL met or exceeded the thresholds that triggered an environmental investigation. In MS and OH, a child’s BLL ≥15 μg/dL triggers a mandatory environmental investigation in the primary residence while in MA, a child’s BLL ≥25 μg/dL triggers a mandatory investigation.

*Subsequent Case:* A newly identified lead poisoned case, identified by case managers between 2000 and 2009, whose BLL met or exceeded the thresholds that triggered an environmental investigation for which this is the first occurrence of lead poisoning, recorded at an address in which an index case was identified. A subsequent case was identified at the address not less than 24 months after identification of the index case and could not have been a case in any other address. This algorithm adjusted for time differences between identification of the index case and development of subsequent cases, as well as adjusted for time needed for remediation and re-habitation.

### Sample size calculation

To calculate the sample size needed for each year of inquiry, we first calculated the proportion of confirmed EBLLs for both female and male children younger than 72 months. We used the formula: as P_F_ = C_F_/T_F_ and P_M_ = C_M_/T_M_, where P_F_ and P_M_ were the proportion of EBLLs, C_F_ and C_M_ were the number of confirmed EBLLs, and T_F_ and T_M_ were the total number tested, for female and male children, respectively. The proportion of cases among male and female children were determined separately because evidence suggests male children are more likely than female children to have EBLLs [11]. To obtain a sample size that mirrored the universe of children in the HHLPPP without sampling all children with EBLL in the program, P_F_ and P_M_ were each multiplied by a constant. This constant, based on the total number of cases identified in the total population of children tested in the HHLPPP, provided a sample fraction that mirrored the actual proportion of children with confirmed EBLL among female and male children in this population in a given year. Thus, to calculate the sample of cases among female and male children needed for the inquiry, we used the formula: Case_F_ = P_F_*constant and Case_M_ = P_M_*constant.

### Data abstraction

We obtained the data for this study from CDC’s CBLS, the case management file of selected cases, and from tax assessor files. Following are the specific types of data we collected and their sources.

#### Address data

We obtained information on addresses in which children with confirmed lead poisoning had resided from files maintained at the MS, OH, and MA Departments of Public Health. Study personnel visited each of these grantees to extract data from paper records that would supplement the blood-test data from the CBLS database for selected children. After children with lead poisoning were identified, random numbers were assigned to each eligible child by gender and year, to facilitate random selection. All dwelling information of these randomly selected children was then used to obtain address level information from the case management and environmental investigation files.

Briefly, auto-generated lists of randomly selected cases were presented to the grantees for file selection who sequentially selected cases based on the random numbers generated. The grantee started at the top of each year or gender grouping and selected available files until the targeted number of cases was selected. If a file was available and selected for a given year, that file became a case patient in that year. To ensure enough cases would be selected, the targeted number of cases in each year and gender combination was increased by three before the list was provided to the grantees. After case patients were selected, their addresses were linked within the case-management file system. This link allowed grantees to obtain information on all addresses at which a child resided at the time of the blood-lead test and case-management follow-up. Information obtained included the year the dwelling was built, who owned the dwelling, and indicators of the presence of lead hazards in the dwelling.

#### Housing/Socio-demographic data

US Census data on population and household characteristics at the county level were downloaded from the US Census Bureau website. These data represented “supplemental data” that helped to better characterize the housing stock and surrounding neighborhoods of addresses and households containing children with data from blood tests. This information was linked to child dwelling records based on the county FIPS code. The sources of the downloaded Census data were Year 2000 Summary Files 1 and 3, available from the following links: http://www2.census.gov/census_2000/datasets/Summary_File_1/ and http://www2.census.gov/census_2000/datasets/Summary_File_3/. The Census data were downloaded for each of the three participating states, and SAS version 9.3 programs were used to analyze the data. County level data were obtained by subsetting by Summary Level 050.

Counties were classified according to their urbanicity using the 2003 Rural–urban Continuum Codes established by the Economic Research Service (ERS) of the U.S. Department of Agriculture (USDA). These codes, available from the USDA website (http://www.ers.usda.gov/data-products/state-fact-sheets/state-data.aspx#Pdc1396023e994cf8868573ebb3245566_2_39iT0), classify each US county (or county equivalent) into one of the nine categories as outlined in Table 3. In assigning codes to counties, a county is considered to be in a metro area if it has at least one urbanized area with population at least 50,000, and either 25% or more of the population of adjacent counties commutes to work in the given county, or 25% or more of workers in these adjacent counties commute from the given county. A county is considered to be in a non-metro area otherwise. Both tax-assessor data and case-management records provided information on whether the dwelling was owner occupied or rented, and the year the dwelling was built. Several counties in OH and MS did not have tax-assessor information readily available online. Tax assessors for these counties were contacted directly for the required information, which was then manually entered into the data-collection system.

**Table 3:** **Summary of building characteristics of addresses with at least one confirmed lead poisoning case** ^**1**^ among resident children with blood-lead measurements collected in 2000 or later for MA and MS, and 2004 or later in OH*

	Number of addresses with children with lead poisoning (% with available responses) ^2^			P-value ^3^
	MA	OH	MS	
Number of addresses	184	216	282	
	**Year building built**			
Pre-1950	28 (47.5%)	121 (87.7%)	74 (57.3%)	*Pre-1950 versus 1950 and newer:*
1950 and newer	3 (5.1%)	1 (0.7%)	2 (1.6%)	
1950 to 1977	4 (6.8%)	13(9.4%)	46 (35.7%)	<0.000 (all states)
Pre-1978	20 (33.9%)	--	--	0.134 (MA vs. MS)
1978 and newer	4 (6.8%)	3 (2.2%)	7 (5.4%)	<0.000 (OH vs. MS)
Unknown	125	78	153	
	**Building type**			
Single-family, detached or attached	58 (43.3%)	97 (70.3%)	121 (74.2%)	< 0.000 (all states)
Multi-unit building	75 (56.0%)	37 (26.8%)	13 (8.0%)	<0.000 (MA vs. MS)
Mobile home	1 (0.8%)	3 (2.2%)	26 (16.0%)	<0.000 (OH vs. MS)
Mix (different categories)	0 (0.0%)	1 (0.7%)	3 (1.9%)	
Unknown	50	78	119	
	**Building ownership**			
Private, owner-occupied	73 (53.3%)	33 (26.0%)	66 (44.3%)	< 0.000 (all states)
Rental, privately-owned	57 (41.6%)	86 (67.7%)	70 (47.0%)	0.098 (MA vs. MS)
Rental, publicly-owned	1 (0.7%)	0 (0.0%)	7 (4.7%)	0.000 (OH vs. MS)
Rental, Section 8 or subsidized	6 (4.4%)	7 (5.5%)	4 (2.7%)	
Mix-both owner-occupied and rental	0 (0.0%)	1 (0.8%)	2 (1.3%)	
Unknown	47	89	133	
	**Building condition**			
Excellent	3 (3.7%)	0 (0.0%)	0 (0.0%)	< 0.000 (all states)
Good	29 (35.8%)	9 (8.1%)	53 (32.3%)	<0.000 (MA vs. MS)
Fair	37 (45.7%)	73 (65.8%)	107 (65.2%)	<0.000 (OH vs. MS)
Poor	10 (12.4%)	29 (26.1%)	1 (0.6%)	
Mix	2 (2.4%)	0 (0.0%)	3 (1.8%)	
Unknown	103	105	118	
	**Prior remodeling or renovation**			
Yes	0 (0.0%)	33 (53.2%)	3 (42.9%)	0.702 (OH vs. MS)
No	0 (0.0%)	29 (46.8%)	4 (57.1%)	
Unknown	184	154	275	
	**Recently repaired or disturbed painted surfaces**			
Yes	43 (65.2%)	36 (52.9%)	1 (100.0%)	0.166 (MA vs. OH)
No	22 (33.3%)	32 (47.1%)	0 (0.0%)	
Both yes and no specified	1 (1.5%)	0 (0.0%)	0 (0.0%)	
Unknown	118	148	281	
	**Intact paint in children’s play areas**			
Yes	24 (42.1%)	13 (46.4%)	17 (73.9%)	0.044 (all states)
No	33 (57.9%)	15 (53.6%)	6 (26.1%)	0.013 (MA vs. MS)
Unknown	127	188	259	0.086 (OH vs. MS)

*Reflects results of the 682 distinct addresses for which at least one confirmed lead poisoning case was identified in which the cohort year was 2000 or later for MA and MS, and 2004 or later for OH.

^1^Defined according to state’s definition as described above.

^2^Percentage reflects the proportion of addresses, among all selected addresses in which at least one confirmed case of lead poisoning was identified, for which this data was available.

^3^Fisher’s Exact test used to examine differences in the distribution of results between states specified in parentheses. P-value ≤0.05 implies statistical significance.

### Analytic strategy

Data were analyzed at the address level. The goal of the analysis was to determine the odds of observing any subsequent lead poisoning cases, in addresses that had on record a previously identified index case, in two states with lead laws compared to one state without lead laws. Thus, an address needed to meet certain eligibility requirements regarding its resident children in order to be included in the analysis. Specifically, an address needed to have at least one child who was eligible to be classified as index case, based on available blood-lead data for the child. For a given address, a child was eligible to be an index case if each of the following occurred:

1. The child was the first child identified at the given address between 2000 and 2009 (or 2004–2009 for OH);
2. The child was not recorded as a case in any other address between 2000 and 2009 (or 2004–2009 for OH) and;
3. The blood lead records for the child are linked to the given address.

In order for subsequent cases to be linked to a specific address, each of the following had to be satisfied:

1. The index case was identified at the address before identification of the subsequent case;
2. The child was not the index case, and;
3. The child was not a confirmed lead poisoning case over the prior two year, the amount of time it takes BLL to decrease once the hazard is removed, while residing at the address or any other address on record.

To examine demographic characteristics of addresses in two states with and one state without laws to prevent childhood lead poisoning, sample means and frequencies were calculated for continuous and categorical socio-demographic, assessed at the county level, and housing variables, respectively. Fisher exact test and Kruskal-Wallis test were used to examine associations in percentages and test for significant differences in mean dust-lead loadings, respectively, between lead law and control states.

A binary variable indicating whether or not any subsequent lead poisoning cases occurred at an address, after identification of an index case was used to define the dependent variable. Since address was the unit of analysis, the binary dependent variable was regressed against the independent variable, lead law state versus non lead law state, to examine whether the odds of observing any subsequent case after identification of an index case differed in addresses in lead law states compared to non-lead law state.

Multivariable logistic regression analyses were used to determine whether this association remained statistically significant after controlling for other risk factors (e.g., socio-demographic, housing, environmental) that a priori had previously been associated independently with EBBLs among young children. The Hosmer-Lemeshow chi-square goodness-of-fit test was performed to determine whether the model was a good fit to the data. The logistic regression model was fitted using the LOGISTIC procedure in SAS.

Institutional Review Board (IRB) approvals were obtained from CDC, the Battelle Memorial Institute, and the MA, MS, and OH Departments of Public Health. All analyses were conducted using SAS version 9.3, SAS Institute, Cary, NC.

## Results

### Address level demographic information

Table 3 summarizes the building characteristics of addresses at which the 623 selected children (i.e., children that could be linked to an address) resided at the time of their blood tests, as recorded in the CBLS database and occurring from 2000 to 2009 in MA and MS, and from 2004 to 2009 in Ohio. To show how frequently children’s primary residence likely changed, and the potential for re-exposure to lead hazards in dwellings that may be unabated, we calculated the number of addresses where a particular child may have lived. On average, the 241 selected children in MA are associated with 1.7 different addresses each, and the 237 children in MS are associated with 2.3 addresses each. In OH, the 145 selected children are associated with an average of 2.1 addresses each.

We analyzed data for the dwelling of a selected child’s primary residence at the time lead poisoning was confirmed, and only for those addresses with associated blood-lead data and evidence of confirmed lead poisoning. MA had 184 addresses in which lead poisoning had been confirmed; OH had 216, and MS had 282. When building age was known, 47.5%, 87.7%, and 57.3% of them in MA, OH, and MS, respectively, were built before 1950. When building type was known, 43.3%, 70.3% and 74.2% of them in MA, OH, and MS, respectively, were single-family dwellings (Table 3). Multi-unit dwellings were more common in MA (56%) than in OH (26.8%) and MS (8.0%). While MA had a greater proportion of private, owner-occupied addresses (53%, versus 26.0% in OH and 44.3% in MS), privately owned rental property was more common in OH (67%) and MS (47%), compared with MA (41.6%). Most of the addresses were listed as being in fair condition, 45.7%, 65.8%, and 65.2% in MA, OH, and MS, respectively (Table 3).

### Primary prevention of childhood lead poisoning

**Figure 1:**
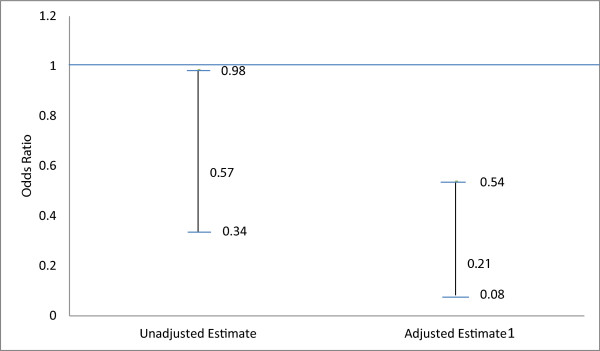
**Unadjusted and adjusted odds ratio and 95% confidence interval of observing addresses with subsequent cases after identification of an index case for lead law states (Massachusetts and Ohio) versus control state (Mississippi).** ^1^Adjusted for building characteristics, environmental factors and county characteristics.

We examined whether statistically significant differences existed, between lead law states and the control state, in the number of confirmed subsequent cases of lead poisoning identified at a given address after the discovery of an index case for that same address. We used logistic regression analysis with the unadjusted and adjusted main effects association expressed as the odds ratios and corresponding 95% confidence interval (CI) (Table 4) (Figure 1). Unadjusted estimates showed that the lead-law states were significantly less likely than the control state to identify subsequent cases of lead poisoning after an index case was identified at a given address, OR = 0.57 95% CI (0.34-0.98) (Figure 1). This association remained statistically significant after controlling for covariates including building characteristics, environmental factors and county characteristics, OR = 0.21; 95% CI (0.08-0.54) (Figure 1). The Hosmer-Lemeshow chi-square value of the goodness-of-fit test was 4.4971, p = 0.480. A p-value less than 0.05 would suggest that the fitted model is not an adequate model; thus given the calculated p-value, there is adequate evidence that the model is a good fit to the data [12].

**Table 4:** **Multiple logistic regression showing unadjusted and adjusted estimates of slope parameters and the odds ratio of observing addresses with subsequent cases after identification of an index case, for lead law states (Massachusetts and Ohio) versus control states (Mississippi)**

Covariate	# Addresses ^1^	Effect Estimates			
		Lead law states (MA and OH) vs. control state (MS)			Odds ratio of lead law states (MA/OH) vs. control state (MS) (95% CI) ^2^
		Estimate	Std. error	p-value*	
Address with subsequent case(s) (Unadjusted main effects model)	292	-0.5546	0.2749	0.0434	0.57 (0.34-0.98)
		**Adjusted estimates**			
Address with subsequent case(s) (Adjusted e stepwise regression main effects association with all variables controlled for in the model)^3,4^	115	-1.5626	0.4806	0.001	0.21 (0.08-0.54)
**Covariate**					
Year building built (pre-1950 vs. newer)	150	-1.3864	0.4090	0.001	
Building type (Single family vs. Multi-unit)	182	-0.7102	0.3642	0.051	
Building ownership (Private, owner-occupied vs. other)	184	-0.8670	0.3440	0.012	
Floor Dust-Lead Loading (mean)	191	-0.6696	0.3283	0.041	
Sill Dust-Lead Loading (mean)	171	-0.9873	0.3469	0.004	
Median Household Income in County (median)	292	-0.6559	0.3945	0.096	
Mean Household Size in County (mean)	292	-0.8508	0.3485	0.015	
Poverty in County (%)	292	-0.4180	0.4044	0.301	
CAPI in County (%)	292	-0.5805	0.2988	0.052	
Households in County with High School Graduates (%)	292	-0.7308	0.4004	0.068	
Non-whites in County (%)	292	-0.5961	0.4144	0.150	
Pre-1950 homes in County (%)	292	-0.3011	0.5540	0.587	
Rentals in County (%)	292	-0.5503	0.2758	0.046	

^1^#addresses represent 292 distinct addresses that had sufficient blood lead data for assessing the potential for subsequent cases following the index case. For the adjusted estimates, the n next to each covariate represents the number of addresses for which that information was available. For the final adjusted model, the N represents the addresses that had both main effects and covariate information available.

^2^Odds ratios are calculated as the exponential of the corresponding slope parameter estimates in this table. Lead law state = 1 and control state = 0.

^3^Results presented shows the final stepwise model adjusting for all covariates listed; the main effects variable, address with subsequent cases, continued to be the best fit to the data. Intercept and county indicator parameters were forced into the stepwise model.

^4^The Hosmer-Lemeshow chi-square value of the goodness-of-fit test was 4.4971, p = 0.4803. Model is a good fit to the data, given p > 0.05.

*P-value ≤0.05 implies statistical significance.

## Discussion

Several studies have been conducted to determine the effectiveness of education and environmental intervention on primary prevention of lead poisoning among infants and young children [13–16]. Only one study [17] has examined the effectiveness of state mandated laws aimed at primary prevention of lead poisoning by preventing lead exposure among children younger than 72 months. In that study the investigators found addresses in limited enforcement areas were 4.6 times more likely to identify at least one subsequent child with blood lead levels of 10 μg/dL or greater compared to strict enforcement areas. This association remained even after controlling for covariates. In this inquiry, we examined the effectiveness of laws aimed at primary prevention of lead poisoning in two states, MA and OH, compared with a state that did not have similar laws, MS, by assessing the likelihood of addresses in the lead law states having subsequent cases of lead poisoning after identification of an index case compared to the likelihood of such observation in the state without lead laws.

Unadjusted estimates showed, compared to the state without lead laws, the lead law states were 43% less likely to have residential addresses with subsequent lead poisoning cases after identification of an index case, among children younger than 72 months. After controlling for covariates including housing and county level risk factors, the association became even stronger, suggesting confounding biased the results towards the null; lead law states were 79% less likely to identify subsequence cases of lead poisoning. Both unadjusted and adjusted results were statistically significant. The MA Lead Law is more stringent and has been enforced much longer than similar laws in OH; however, evidence exists that when any lead law is enacted and enforced, they can be effective in reducing primary exposure to lead among young children [17]. The evidence here may confirm the assertion that enforced lead laws, regardless of their stringency, can effectively decrease subsequent lead poisoning among children younger than 72 months.

Potential beneficiaries of laws aimed at preventing lead poisoning may be families re-gentrifying inner city neighborhoods [15] since the demographics of families living in homes with potential lead-poisoning hazards are changing. Historically lead poisoning has been considered a public health problem found primarily among young children in families whose head of household was often less educated and had lower income; we now find a changing structure in many inner city neighborhoods [18]. Where once dilapidated, single-family, older homes stood, often the victim of urban blight, we find newly remodeled structures inhabited by more affluent families [18].

The results of these findings are important and warrant further research given recent recommendations from CDC’s Advisory Committee on Childhood Lead Poisoning Prevention (ACCLPP) to lower the blood-lead level at which public health action is taken. Between 1991 and 2011, CDC defined BLLs ≥10 *μ*g/dL as the “level of concern” for children aged 1–5 years [19]. However, in May 2012, CDC accepted its ACCLPP recommendations that use of the term “level of concern” be discontinued and replaced. This replacement term will be the upper value of a reference range, calculated from two consecutive cycles from the National Health and Nutritional Examination Survey (NHANES), defined as the 97.5th percentile of BLLs among U.S. children aged 1–5 years [20]. Based on NHANES estimates, from the combined 2007–2008 and 2009–2010 data cycles, the 97.5th percentile distribution of BLL among U.S. children aged 1–5 years is 5 *μ*g/dL [20].

While efforts were made to control for the effect of confounding and limit sampling bias, the results of this study are not without limitations. Unavailable demographic and environmental data, at the address level, as well as residual confounding at the individual level, may have limited our ability to thoroughly control for the effect of external factors, which may cause deceptive associations. To confirm whether there may have been residual confounding due to missing address level data, ancillary analyses were conducted to determine whether differences existed in median county level pre-1950’s homes, household income and poverty levels among addresses with complete address level information compared to those with missing information. There was no statistically significant difference in median county level pre-1950’s homes, household income and poverty level between addresses with and without complete address level information.

Limitation due to case attrition may have affected our ability to depict true exposure experiences of patients who subsequently were diagnosed with lead poisoning, the result of which would have diluted the true association. Additionally, while efforts were made to control for the effect of confounding at the county level, as was detailed in Table 4 where we controlled for county level poverty, pre-1950’s housing, and median household income to name a few, there may have been other unmeasured county level risk factors which may have resulted in spurious associations, for example ecological confounding.

The effects of ecological confounding at the state level may have limited our ability to delineate true associations. For example, the state without lead laws, tended to on average, have more privately owned rental and owner occupied addresses compared to the lead law states. Privately owned property, built before 1978, is not federally mandated to abate residential lead paint [21]. Thus, ecological confounding due to ownership status may have confounded the main effects association, biasing our results towards the null. Such findings were confirmed with our adjusted main effects estimate which became stronger after controlling for confounding at the address level. That notwithstanding, the results of this study do show an association between the likelihood of an address having a subsequent case of lead poisoning after identification of an index case and the presence or absence of state lead laws.

## Conclusions

Although all three states may have dwellings with lead-paint hazards, the results of this study suggests that compared to MS, laws in MA and OH may be effective in reducing the number of young children exposed to residential lead contamination. This study has shown that compared to children living in the one state examined without lead laws, children younger than 72 months living in the two states with lead laws are less likely to live at an address where a previous child was found to be lead poisoned and become a subsequent case. This evidence suggests that compared to the state with lead laws, laws such as those studied herein can reduce exposure to residential lead contamination among young children, given the fewer number of subsequent cases of lead poisoning identified at residential addresses where a previously detected index case was recorded.

## Abbreviations

CDC: Centers for Disease Control and Prevention
CLPPP: Childhood Lead Poisoning and Prevention Program
CBLS: Childhood Blood Lead Surveillance
EBLL: Elevated Blood Lead Level
HHLPPP: Healthy Homes and Lead Poisoning Prevention Program
MA: Massachusetts
MS: Mississippi
OH: Ohio.
